# Supplementary material for: The Systems Biology Research Tool: evolvable open-source software
Source: BMC Syst Biol. 2008 Jun 29;2:55. doi: 10.1186/1752-0509-2-55 (PMC2446383; doi:10.1186/1752-0509-2-55)
Supplement: Additional file 1 — SBRT Archive. An archive of the current version of the Systems Biology Research Tool. [file 1752-0509-2-55-S1.zip › sbrt-1.4.0/doc/developers_guide/api/sbrt/kernel/math/Polynomial.Term.html]

Polynomial.Term


|  |  |  |
| --- | --- | --- |
| **Overview** \| **Package** \| **Class** \| **Use** \| **Tree** \| **Deprecated** \| **Index** \| **Help** | |  |
| **PREV CLASS**   **NEXT CLASS** | **FRAMES**    **NO FRAMES**     **All Classes** |  |
| SUMMARY: NESTED \| FIELD \| CONSTR \| METHOD | DETAIL: FIELD \| CONSTR \| METHOD |  |


---


## sbrt.kernel.math Interface Polynomial.Term<V>

**Type Parameters:**: `V` - the variable type.

**All Superinterfaces:**: java.lang.Iterable<V>

**All Known Subinterfaces:**: LinearComb.Term<V>, QuadraticExpr.Term<V>

**All Known Implementing Classes:**: AbstractLinearComb.Term, HashLinearComb.Term

**Enclosing interface:**: Polynomial<V>

---

``` public static interface Polynomial.Term<V> extends java.lang.Iterable<V> ```

This interface is used to represent the terms of a
polynomial. A term consists of a numerical coefficient
and a set of variables, with each variable raised to
a non-negative whole number power.

**Author:**
:   This interface was written and documented by
    Jeremiah Wright while in the Wagner lab.

---

| **Method Summary** | |
| --- | --- |
| `boolean` | `equals(java.lang.Object obj)`             Indicates if the provided object is equal to this term. |
| `double` | `getCoefficient()`             Returns the coefficient of this term. |
| `java.util.Set<V>` | `getVariables()`             Returns the set of variables contained in this term. |
| `boolean` | `hasAllVariables(java.util.Collection<?> variables)`             Indicates if this term contains all of the variables in the provided collection. |
| `int` | `hashCode()`             Returns a content-based hash code for this term. |
| `boolean` | `hasVariable(java.lang.Object variable)`             Indicates if the provided object is a variable contained in this term. |
| `java.util.Iterator<V>` | `iterator()`             Returns an iterator over the variables in this term. |
| `int` | `power(java.lang.Object variable)`             Returns the power of the provided variable in this term. |
| `java.lang.String` | `toString()`             Returns an appropriate string representation of this term. |
| `int` | `variables()`             Returns the number of variables contained in this term. |

**Method Detail**

### getCoefficient

```
double getCoefficient()
```

:   Returns the coefficient of this term.

    :   **Returns:**: the coefficient of this term.

---


### iterator

```
java.util.Iterator<V> iterator()
```

:   Returns an iterator over the variables in this term.

    :   **Specified by:**: `iterator` in interface `java.lang.Iterable<V>`
    :   **Returns:**: an iterator over the variables in this term.

---


### power

```
int power(java.lang.Object variable)
```

:   Returns the power of the provided variable in this
    term.

    :   **Parameters:**: `variable` - the variable whose power is to be returned. **Returns:**: the power of the provided variable in this term. If the provided object is not a variable in this term, 0 is returned.

---


### getVariables

```
java.util.Set<V> getVariables()
```

:   Returns the set of variables contained in this term.

    :   **Returns:**: the set of variables contained in this term.

---


### variables

```
int variables()
```

:   Returns the number of variables contained in this term.

    :   **Returns:**: the number of variables contained in this term.

---


### hasVariable

```
boolean hasVariable(java.lang.Object variable)
```

:   Indicates if the provided object is a variable contained
    in this term.

    :   **Parameters:**: `variable` - the variable whose presence in this term is to be tested. **Returns:**: `true` if this term contains the provided variable; `false` otherwise.

---


### hasAllVariables

```
boolean hasAllVariables(java.util.Collection<?> variables)
```

:   Indicates if this term contains all
    of the variables in the provided collection.

    :   **Parameters:**: `variables` - the collection of variables whose presence in this term is to be tested. **Returns:**: `true` if each element of the provided collection is a variable in this term; `false` otherwise.

---


### hashCode

```
int hashCode()
```

:   Returns a content-based hash code for this term.

    :   **Overrides:**: `hashCode` in class `java.lang.Object`
    :   **Returns:**: a content-based hash code for this term.

---


### equals

```
boolean equals(java.lang.Object obj)
```

:   Indicates if the provided object is equal to this
    term.

    Two terms are considered equal if they 1) contain
    the same coefficient, 2) contain the same variables,
    and 3) the powers of each variable are the same in
    both terms.

    :   **Overrides:**: `equals` in class `java.lang.Object`
    :   **Parameters:**: `obj` - the object to be tested for equality with this term. **Returns:**: `true` if this term is equal to the provided object; `false` otherwise.

---


### toString

```
java.lang.String toString()
```

:   Returns an appropriate string representation of this
    term.

    :   **Overrides:**: `toString` in class `java.lang.Object`
    :   **Returns:**: an appropriate string representation of this term.


---


|  |  |  |
| --- | --- | --- |
| **Overview** \| **Package** \| **Class** \| **Use** \| **Tree** \| **Deprecated** \| **Index** \| **Help** | |  |
| **PREV CLASS**   **NEXT CLASS** | **FRAMES**    **NO FRAMES**     **All Classes** |  |
| SUMMARY: NESTED \| FIELD \| CONSTR \| METHOD | DETAIL: FIELD \| CONSTR \| METHOD |  |


---
